# Author Correction: Evaluation of molecular subtypes and clonal selection during establishment of patient-derived tumor xenografts from gastric adenocarcinoma

**DOI:** 10.1038/s42003-020-01135-5

**Published:** 2020-07-24

**Authors:** Anne-Lise Peille, Vincent Vuaroqueaux, Swee-Seong Wong, Jason Ting, Kerstin Klingner, Bruno Zeitouni, Manuel Landesfeind, Woo Ho Kim, Hyuk-Joon Lee, Seong-Ho Kong, Isabella Wulur, Steven Bray, Peter Bronsert, Nina Zanella, Greg Donoho, Han-Kwang Yang, Heinz-Herbert Fiebig, Christoph Reinhard, Amit Aggarwal

**Affiliations:** 1 Charles River Discovery Research Services Germany GmbH (formerly Oncotest GmbH), Am Flughafen 12-14, 79108 Freiburg, Germany; 2 Lilly Research Labs, Eli Lilly and Company, Indianapolis, IN 46285 USA; 3 Department of Pathology, Seoul National University College of Medicine, 101 Daehak-ro, Jongnogu, Seoul, Korea; 4 Department of Surgery and Cancer Research Institute, Seoul National University College of Medicine, 101 Daehak-ro, Jongno-gu, Seoul, Korea; 5 Department of Surgery, Seoul National University Hospital, 101 Daehak-ro, Jongno-gu, Seoul, Korea; 6 Institute for Surgical Pathology, Medical Center—University of Freiburg, Freiburg, Germany; 7 Comprehensive Cancer Center Freiburg, Medical Center—University of Freiburg, Freiburg, Germany; 8 Faculty of Medicine, University of Freiburg, Freiburg, Germany; 9 Present Address: 4HF Biotec GmbH, Am Flughafen 14, Freiburg, 79108 Germany; 10 Present Address: LifeOmic, 351 W 10th St, Indianapolis, IN USA; 11 Present Address: Evotec International GmbH, Marie-Curie-Strasse, 37079 Göttingen, Germany

**Keywords:** Cancer models, Gastric cancer, Cancer genetics

Correction to: *Communications Biology*10.1038/s42003-020-1077-z, published online 9 July 2020.

The original version of this Article contained an error in the spelling of the author Kerstin Klingner, which was incorrectly given as Kerstin Klinger. This has now been corrected in both the PDF and HTML versions of the Article.

