# Supplementary material for: Evaluation of molecular subtypes and clonal selection during establishment of patient-derived tumor xenografts from gastric adenocarcinoma
Source: Commun Biol. 2020 Jul 9;3:367. doi: 10.1038/s42003-020-1077-z (PMC7347869; doi:10.1038/s42003-020-1077-z)
Supplement: Supplementary file 1 — Supplementary Information [file 42003_2020_1077_MOESM1_ESM.pdf]

Supplementary Information

Supplementary figures

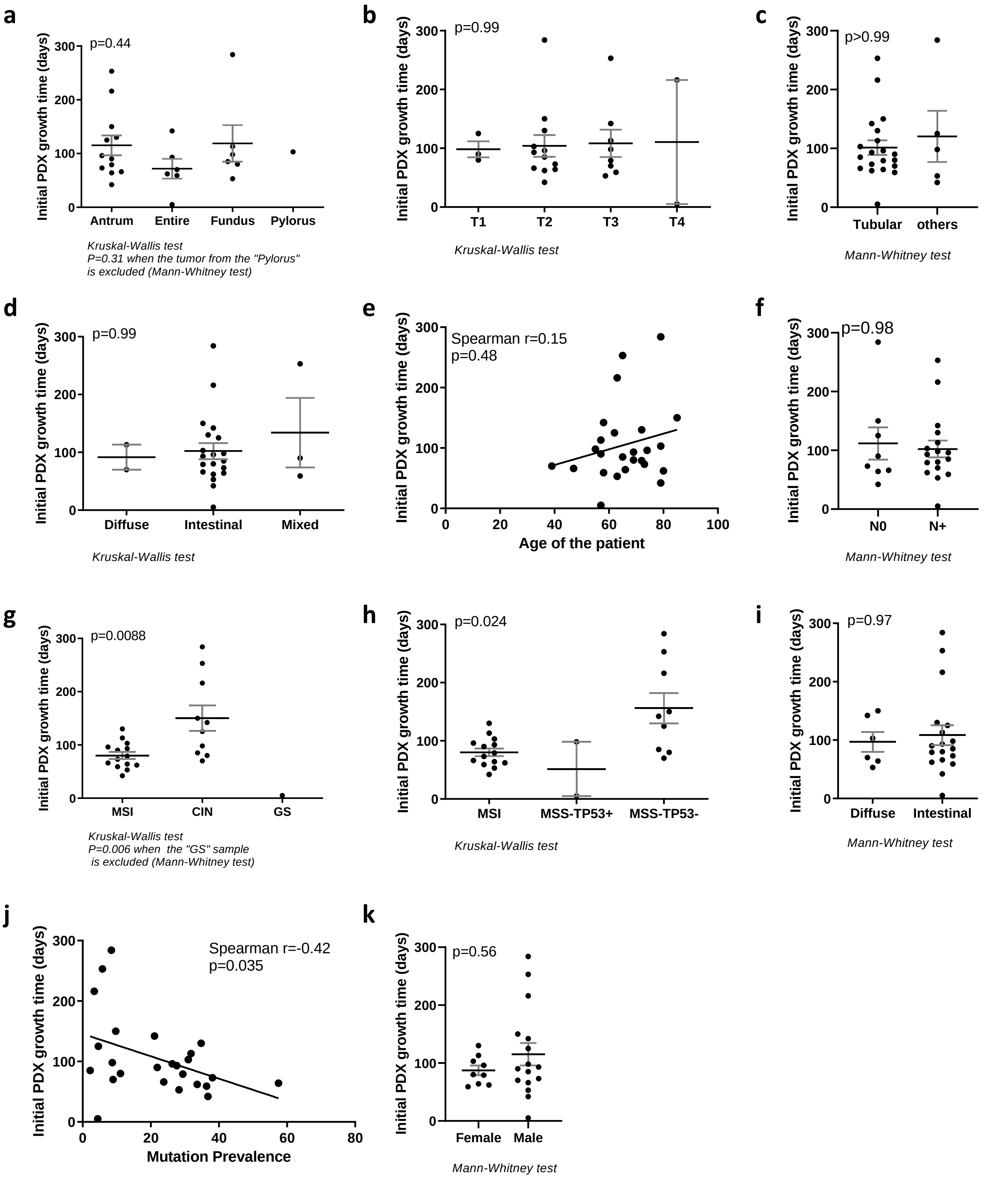

Supplementary Fig. 1 Parameters associated with the tumor growth at initial implantation of the patient tumors on immune-compromised mice. Comparison between the tumor growth in days and **a.** the localization of the primary tumors, **b.** the tumor stage, **c.** the localization according to the WHO classification, **d.** the Lauren subtypes of the established PDX, **e.** the age of the patients at surgery, **f.** the lymph node status, **g.** the TCGA subtypes, **h.** the ACRG subtypes, **i.** the Lauren subtypes of the primary tumors, **j.** the mutation prevalence, **k.** the gender of the patients. The black bars represent the mean and the grey bars the standard error of the mean.

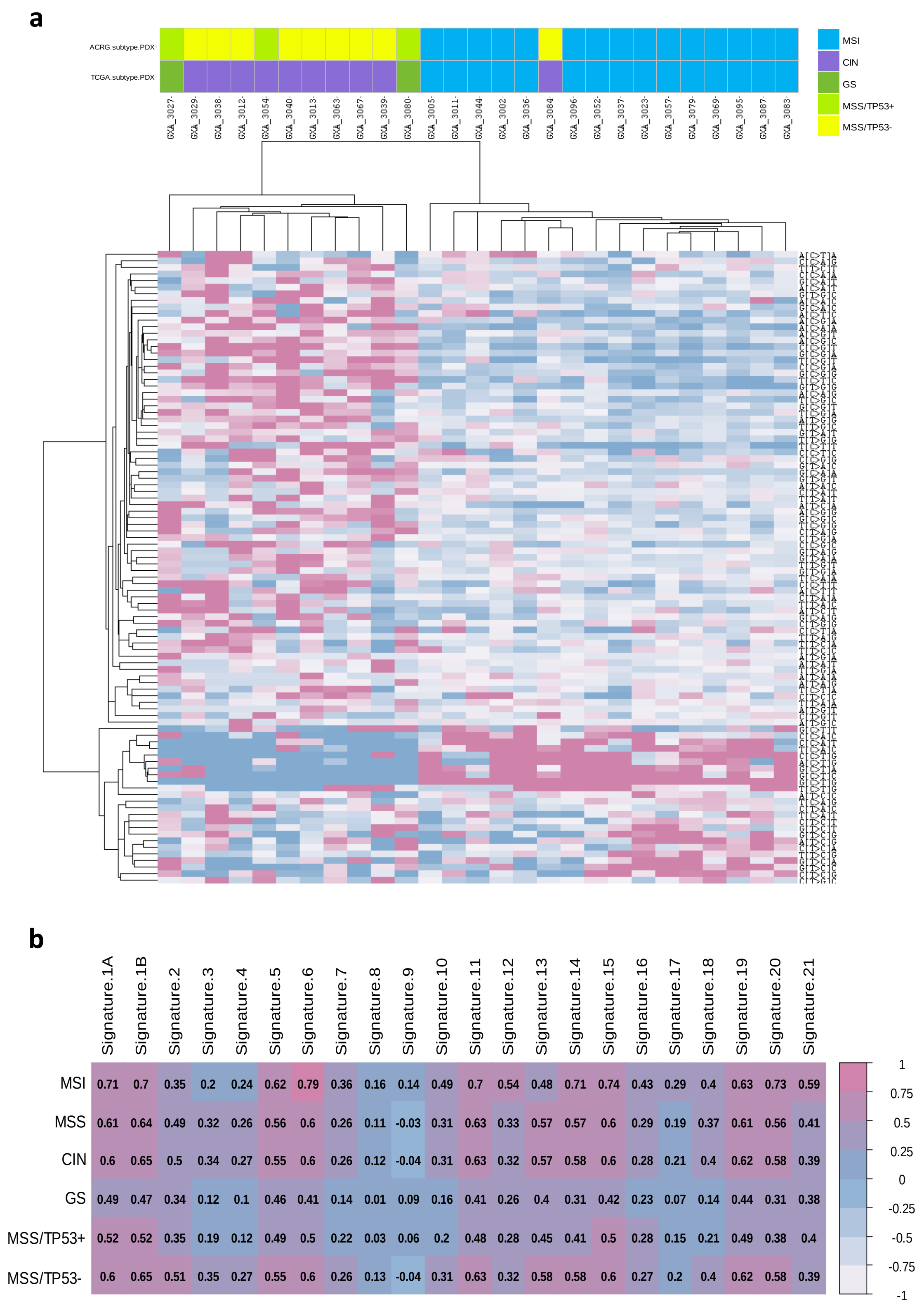

Supplementary Fig. 2 mutational signatures of gastric cancer PDX. a. Unsupervised hierarchical clustering of the proportions of the 96 possibilities of nucleotide substitutions (in context of the 3’ 5’ nucleotides flanking the mutated base) within the 27 PDX. Molecular subtypes of the PDX according to the TCGA and ACRG classification are also indicated. **b.** Correlation matrix of the PDX signatures of mutational processes and the signatures of mutational processes published in patient tumors by Alexandrov et al,. Spearman correlation values are indicated in black.

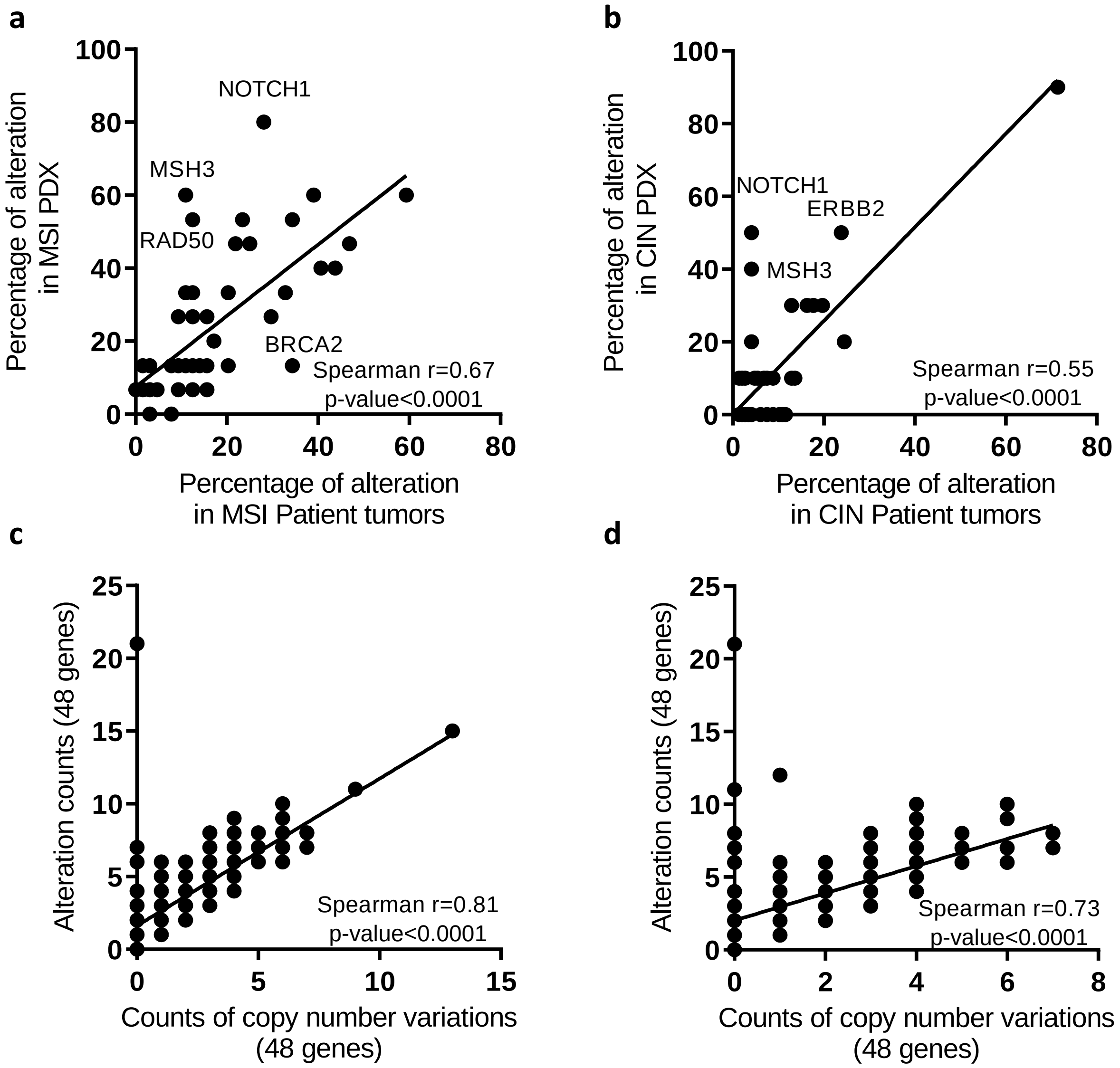

Supplementary Fig. 3 Correlation between the percentages of alterations in the 48 potentially targetable cancer genes observed in the PDX collection and in the gastric cancer patient tumors from the TCGA. **a.** The percentages were analyzed in the MSI subtype (15 PDX and 64 patient tumors). **b.** The analysis was done in CIN subtype (10 PDX and 147 patient tumors). **c.** Counts of gene copy number alterations compared to the total alteration counts for the 48 potentially targetable genes in the MSS patient tumor samples as defined by the TCGA. **d.** Counts of gene copy number alterations compared to the total alteration counts for the 48 potentially targetable genes in the MSS samples as defined by the ACRG.

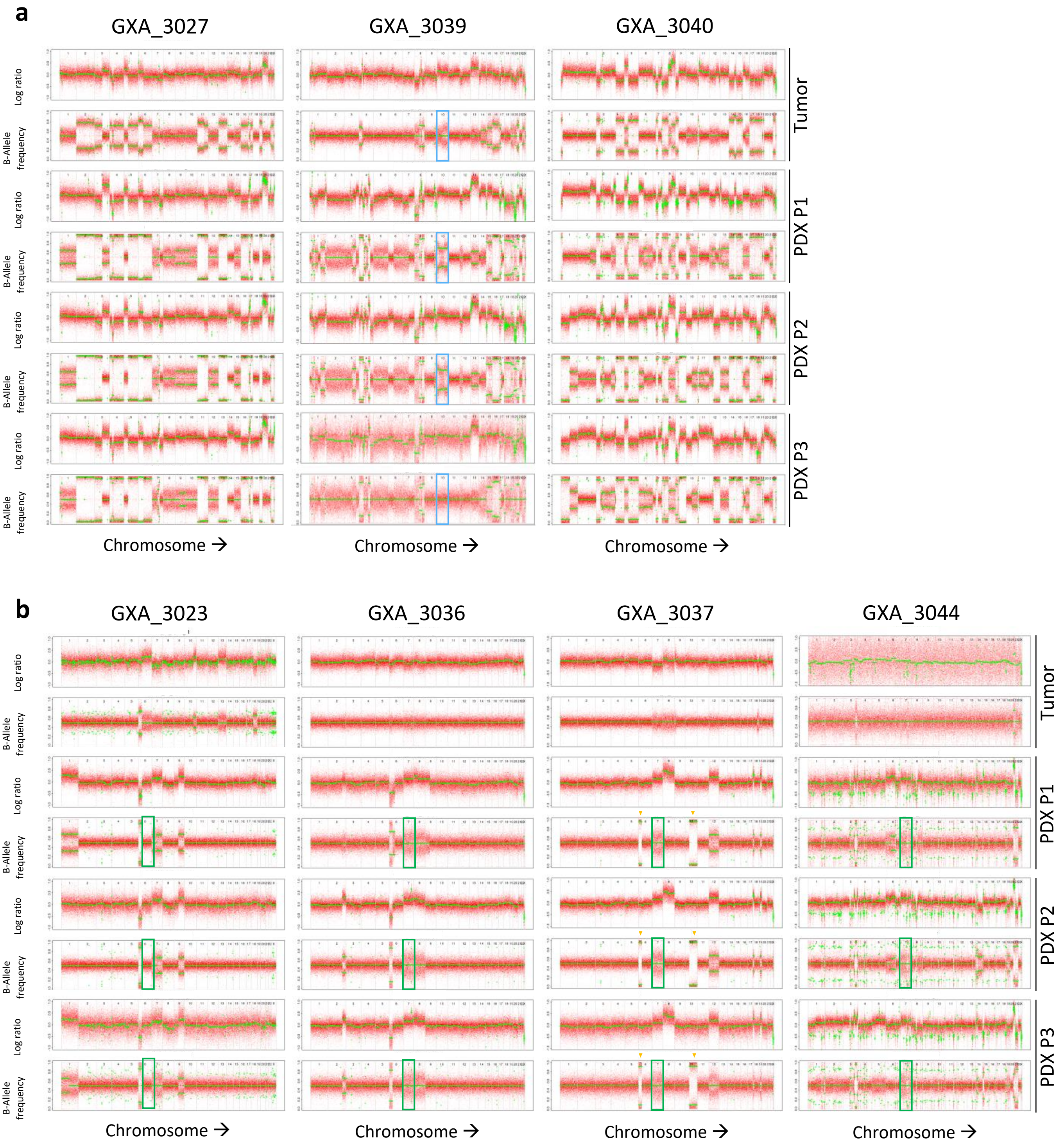

Supplementary Fig. 4 Gastric cancer PDX clonal variation through passages. a. Log ratio and B-allele frequency plots from Affymetrix SNP6.0 arrays for the patient tumor and the PDX at passage 1, 2 and 3 (P1, P2, P3) of the MSS series (GXA_3027, GXA_3039, GXA_3040), show chromosomal changes in copy number. **b**. Plots for the four MSI series (GXA_3023, GXA3036, GXA 3037 and GXA_3044) not shown here displayed a normal diploid profile with sparse alterations. One piece of each tumor sample was analyzed per condition. Result for GXA_3038 is not available.

Supplementary Tables

Supplementary Table 1: Primers used in qRT-PCR and PCR

Official Gene Symbol	Gene Title	Primer F sequence (5'-3')	Primer R sequence (5'-3')	Annealing Temperature	Amplicon Size (bp)	Primers used for
EBNA1	Epstein–Barr virus nuclear antigen 1	GCCGGTGTGTTCGTATATGG	CAAAACCTCAGCAAATATATGAG	60°C	213	EBV status
MLH1	mutL homolog 1	CTACTTCCAGCAACCCCAGA	CTTTCGGGAATCATCTTCCA	60°C	74	MLH1 mRNA expression
RN18S5	RNA, 18S ribosomal 5	CTACCACATCCAAGGAAGGCA	TTTTTCGTCACTACCTCCCCG	60°C	71	18s expression
ERBB2	erb-b2 receptor tyrosine kinase 2	CTGAACTGGTGTATGCAGATTGC	TTCCGAGCGGCCAAGTC	60°C	220	ERBB2 gene copy number
TOP2A	topoisomerase (DNA) II alpha	CAACATGCCAATTGAGTGAAA	ACTTGGGCCTTAAACTTCACC	60°C	94	TOP2A mRNA expression
MDM2	Mdm2 p53 binding protein homolog	AAAGAGCACAGGAAAATATATACCATGA	GGTGACACCTGTTCTCACTCACA	60°C	106	MDM2 mRNA expression
CDH1	cadherin 1, type 1, E-cadherin	GCATTGCCACATACACTCTCTTCT	GATCGGTTACCGTGATCAAAATC	60°C	93	CDH1 mRNA expression
CDKN1A	Cyclin kinase Inhibitor (p21)/cyclin-dependent kinase inhibitor 1A (p21, Cip1)	GCAGACCAGCATGACAGATTTC	GCGGATTAGGGCTTCCTCTT	60°C	72	CDKN1A mRNA expression
MKI67	antigen identified by monoclonal antibody Ki-67	TGCCTTGGTCTCTTGGGAAT	TAGGAGCCAGTTTGAGGTCG	60°C	118	MKI67 mRNA expression
GAPDH	glyceraldehyde-3-phosphate dehydrogenase	CAAATTCCATGGCACCGTC	GCCACACCATCCTAGTTGC	60°C	133	GAPDH gene copy number
MLH1	mutL homolog 1	GAGGCTTTGGCCAGCATAA	TGAGGTACAGGAATGGGTGTG	61°C	580	MLH1 mutation status (mRNA)
MLH1	mutL homolog 1	CAACTTCCTTGAGAAAAGCCATA	GACATTTCTGAAGTCCCCTTTG	60°C	571	MLH1 mutation status (mRNA)
MLH1	mutL homolog 1	AAGTGGCTGCCAAAAATCAG	CCCTTCCTCATCAATTTCCA	60°C	587	MLH1 mutation status (mRNA)
MLH1	mutL homolog 1	CCCAAAGAAGGACTTGCTGA	GGCAAGTATAAGTCTTAAGTGCTACC	60°C	594	MLH1 mutation status (mRNA)
MLH1	mutL homolog 1	CAAAATGTCGTTCGTGGCAG	GTGCCATTGTCTTGGATCTG	60°C	196	MLH1 mutation status (mRNA)
MLH1	mutL homolog 1	GTGATTGTTAAAGAGGGAGGC	TTGCTCTGTATGCACACTTTCC	60°C	241	MLH1 mutation status (mRNA)
MLH1	mutL homolog 1	GCTGAAGGAAGAACGTGAGC	CTCCCTCCGTACCAGTTCTC	62°C	224	MLH1 mutation status (DNA)

Supplementary Table 2: statistics of whole exome sequencing data of the 27 gastric cancer PDX

Tumor name	Enrichment Kit	Mapped reads on targets	Coverage on targets	Human Map Better Read%	Mouse Map Better Read%
GXA_3002	Agilent V1 38MB	77.60%	101.73	94.67	5.22
GXA_3005	Agilent V1 38MB	77.82%	99.36	94.30	5.60
GXA_3011	Agilent V4 51MB	75.95%	158.1	92.70	7.18
GXA_3012	Agilent V4 51MB	75.76%	122.19	93.78	6.05
GXA_3013	Agilent V1 38MB	77.70%	100.16	97.14	2.77
GXA_3023	Agilent V1 38MB	79.41%	110.56	95.94	3.97
GXA_3027	Agilent V1 38MB	78.93%	100.39	95.77	4.14
GXA_3029	Agilent V4 51MB	74.54%	109.71	96.59	3.32
GXA_3036	Agilent V4 51MB	78.55%	124.85	82.61	16.84
GXA_3037	Agilent V4 51MB	75.26%	125.08	88.14	11.64
GXA_3038	Agilent V4 51MB	77.33%	179.71	95.30	4.55
GXA_3039	Agilent V4 51MB	77.81%	114.25	90.30	9.27
GXA_3040	Agilent V4 51MB	74.75%	131.23	95.01	4.80
GXA_3044	Agilent V4 51MB	77.52%	115.03	78.72	21.05
GXA_3052	Agilent V4 51MB	75.19%	130.2	94.63	4.90
GXA_3054	Agilent V4 51MB	75.57%	114.23	98.14	1.77
GXA_3057	Agilent V4 51MB	75.25%	113.48	98.02	1.91
GXA_3067	Agilent V4 51MB	75.98%	153.52	98.04	1.68
GXA_3063	Agilent V5 50MB	74.63%	105.1	93.65	2.81
GXA_3083	Agilent V5 50MB	75.63%	122.3	91.01	4.54
GXA_3087	Agilent V4 51MB	76.84%	153.85	92.60	6.64
GXA_3069	Agilent V4 51MB	71.50%	118.94	97.03	2.62
GXA_3095	Agilent V4 51MB	73.84%	153.14	92.26	6.91
GXA_3079	Agilent V4 51MB	73.36%	143.86	93.58	5.76
GXA_3080	Agilent V4 51MB	76.57%	181.36	98.16	1.59
GXA_3084	Agilent V4 51MB	75.80%	215.62	96.49	3.12
GXA_3096	Agilent V4 51MB	73.80%	130.32	97.76	1.93

Supplementary Table 3: Primers and probes used in droplet digital PCR mutation assays.

Assay	Forward Primer	Reverse Primer	WT Probe	Mutant Probe	Manufactur-er
KRAS G13D (c.38G>A)	CTGAAAATGACTGAATATAAACTTGTGG	TAGCTGTATCGTCAAGGCACTC	VIC-CTGGTGGCGTAGGC-NFQ	6FAM-CTGGTGACGTAGGC-NFQ	Life Tech
PIK3CA H1047R (c.3140A>G)	GAGCAAGAGGCTTTGGAGTA	ATGCTGTTTAATTGTGTGGAAGA	/5TET/C+CATG+A+T+GT/ZEN/G+CAT/3IABkFQ/	/56-FAM/C+CATG+A+C+GT/ZEN/GCAT/3IABkFQ/	IDT
PIK3CA G1049R (c.3145G>C)	GCAAGAGGCTTTGGAGTATTTCATG	GCATGCTGTTTAATTGTGTGGAAGA	6FAM-TTGTCCAGCCACGATGA-NFQ	VIC-TTGTCCAGCCACCATGA-NFQ	Life Tech
PIK3CA R88Q (c.263G>A)	GTTACTCAAGAAGCAGAAAGGGAAGA	AGGGTTGAAAAAGCCGAAGGT	VIC-ATGAAACAAGACGACTTTG-NFQ	6FAM-AAACAAGACAACTTTG-NFQ	Life Tech
