# Supplementary material for: Evaluation of molecular subtypes and clonal selection during establishment of patient-derived tumor xenografts from gastric adenocarcinoma
Source: Commun Biol. 2020 Jul 9;3:367. doi: 10.1038/s42003-020-1077-z (PMC7347869; doi:10.1038/s42003-020-1077-z)
Supplement: Supplementary file 2 — Description of Additional Supplementary Files [file 42003_2020_1077_MOESM2_ESM.pdf]

Description of additional supplementary files

Supplementary Data (excel)

Contains Supplementary Data-sets 1-15
