# Supplementary material for: Evaluation of molecular subtypes and clonal selection during establishment of patient-derived tumor xenografts from gastric adenocarcinoma
Source: Commun Biol. 2020 Jul 9;3:367. doi: 10.1038/s42003-020-1077-z (PMC7347869; doi:10.1038/s42003-020-1077-z)
Supplement: Supplementary file 4 — Reporting Summary [file 42003_2020_1077_MOESM4_ESM.pdf]

nature research

Corresponding author(s): Heinz-Herbert Fiebig, Christoph Reinhard, Amit Aggarwal

Last updated by author(s): Apr 26, 2020

Reporting Summary

Nature Research wishes to improve the reproducibility of the work that we publish. This form provides structure for consistency and transparency in reporting. For further information on Nature Research policies, see Authors & Referees and the Editorial Policy Checklist.

Statistics

For all statistical analyses, confirm that the following items are present in the figure legend, table legend, main text, or Methods section.

n/a	Confirmed	
☐	☒	The exact sample size (*n*) for each experimental group/condition, given as a discrete number and unit of measurement
☐	☒	A statement on whether measurements were taken from distinct samples or whether the same sample was measured repeatedly
☐	☒	The statistical test(s) used AND whether they are one- or two-sided<br>*Only common tests should be described solely by name; describe more complex techniques in the Methods section.*
☒	☐	A description of all covariates tested
☒	☐	A description of any assumptions or corrections, such as tests of normality and adjustment for multiple comparisons
☒	☐	A full description of the statistical parameters including central tendency (e.g. means) or other basic estimates (e.g. regression coefficient) AND variation (e.g. standard deviation) or associated estimates of uncertainty (e.g. confidence intervals)
☐	☒	For null hypothesis testing, the test statistic (e.g. *F*, *t*, *r*) with confidence intervals, effect sizes, degrees of freedom and *P* value noted<br>*Give P values as exact values whenever suitable.*
☒	☐	For Bayesian analysis, information on the choice of priors and Markov chain Monte Carlo settings
☒	☐	For hierarchical and complex designs, identification of the appropriate level for tests and full reporting of outcomes
☒	☐	Estimates of effect sizes (e.g. Cohen's *d*, Pearson's *r*), indicating how they were calculated

Our web collection on statistics for biologists contains articles on many of the points above.

Software and code

Policy information about availability of computer code

Data collection	we used the software R (version 2.15.3) and GraphPad Prism version 5. All described in the material and method section
Data analysis	we used custom R codes for most of the statistical analyses. Source codes are available upon request (email the corresponding authors)

For manuscripts utilizing custom algorithms or software that are central to the research but not yet described in published literature, software must be made available to editors/reviewers. We strongly encourage code deposition in a community repository (e.g. GitHub). See the Nature Research guidelines for submitting code & software for further information.

Data

Policy information about availability of data

All manuscripts must include a data availability statement. This statement should provide the following information, where applicable:

- Accession codes, unique identifiers, or web links for publicly available datasets
- A list of figures that have associated raw data
- A description of any restrictions on data availability

The molecular data of the 27 established Asian gastric PDX can be queried on the Charles River Tumor Model Compendium at “https://compendium.criver.com”. The whole exome sequencing data (raw FASTQ files) of the PDX models has been deposited in SRA (Sequence Read Archive) under the accession code SRP150675. The raw (CEL files) Affymetrix HGU133 Plus 2.0 transcriptomic data of the 27 Asian gastric PDX models that support the results presented in this paper has been deposited in GEO (Gene Expression Omnibus) under the accession code GSE115637. The raw (CEL files) Affymetrix SNP6.0 data and the PICNIC processed genomic data presented in this study for the 27 established Asian gastric PDX models, 7 normal tissues, 7 patient tumors and 21 PDX samples at passages 1, 2 and 3 have been deposited in GEO under the accession code GSE115674. All Affymetrix data can be accessed via the GEO code GSE115755. The molecular data of the 295 patient tumors from the TCGA cohort (TCGA Nature, 2014, DOI: 10.1038/nature13480) and the associated clinical data are accessible from the cBioPortal (http://www.cbioportal.org/study/summary?id=stad_tcga_pub). The ACRG subtypes of the 295 gastric tumors from the TCGA dataset were presented in the paper published by Cristescu et al (Nature Medicine, 2015, doi:10.1038/nm.3850) and are available upon request to Amit Aggarwal (aggarwal_amit@lilly.com).

The PDX samples are the proprietary of Charles River Discovery Services GmbH, Freiburg Germany. The established PDX models can be used for research projects on a fee-for service model. The DNA and RNA samples prepared from the established PDX models are the proprietary of Charles River Discovery Services GmbH, Freiburg Germany and can be purchased on demand.

Field-specific reporting

Please select the one below that is the best fit for your research. If you are not sure, read the appropriate sections before making your selection.

☒ Life sciences ☐ Behavioural & social sciences ☐ Ecological, evolutionary & environmental sciences

For a reference copy of the document with all sections, see nature.com/documents/nr-reporting-summary-flat.pdf

Life sciences study design

All studies must disclose on these points even when the disclosure is negative.

Sample size	Tumors and associated peritumoral normal tissues from 100 Asian patients with gastric cancer were used in this project to develop PDX models. A total of 27 PDX modes were succesfully established. We also included in this study data from the TCGA (stomach adenocarcinoma (TCGA Nature, 2014, 295 tumors)
Data exclusions	none
Replication	none
Randomization	not required
Blinding	not required

Reporting for specific materials, systems and methods

We require information from authors about some types of materials, experimental systems and methods used in many studies. Here, indicate whether each material, system or method listed is relevant to your study. If you are not sure if a list item applies to your research, read the appropriate section before selecting a response.

Materials & experimental systems

n/a	Involved in the study	
☐	☒	Antibodies
☒	☐	Eukaryotic cell lines
☒	☐	Palaeontology
☐	☒	Animals and other organisms
☐	☒	Human research participants
☒	☐	Clinical data

Methods

n/a	Involved in the study	
☒	☐	ChIP-seq
☒	☐	Flow cytometry
☒	☐	MRI-based neuroimaging

Antibodies

Antibodies used	the antibodies anti-human MLH1 and anti-human ERBB2 were used in this study for IHC analyses. The references of the antibodies are indicated in the methods section
Validation	the antibodies and the immunohistochemistry (IHC) protocols were develpped by Roche-Ventana and were used according to the manufacturer´s instructions

Animals and other organisms

Policy information about studies involving animals; ARRIVE guidelines recommended for reporting animal research

Laboratory animals	Female NMRI nude mice obtained from Harlan (Denmark) at the age of 4-6 weeks were used to establish and maintain the PDX models
Wild animals	no
Field-collected samples	no
Ethics oversight	All animal experiments were approved by the Committee on the Ethics of Animal Experiments of the regional council

Ethics oversight	(Regierungspräsidium Freiburg, Abt. Landwirtschaft, Ländlicher Raum, Veterinär- und Lebensmittelwesen - Ref. 35, permit-#: G-13/13).

Note that full information on the approval of the study protocol must also be provided in the manuscript.

Human research participants

Policy information about studies involving human research participants

Population characteristics	Asian patients with gastric cancer
Recruitment	100 patients with gastric cancer. No further criteria used
Ethics oversight	All patients provided informed consent and SNU IRB approved the study (IRB number H-0807-037-250).

Note that full information on the approval of the study protocol must also be provided in the manuscript.
